# Man and Other Animals

**Published:** 1875-12

**Authors:** 


### MAN AND OTHER ANIMALS.

All animals, to be healthy, must have a good house for shelter, plenty of uncontaminated, pure air, and, above all, the glorious, revivifying, life-engendering sunlight. Without the beautiful sun, naught can possibly thrive or be healthy.

Yet, there is such a thing as too much light. We must not allow the direct rays of the sun to strike the page which we read, or upon which we write. We must not whitewash the walls around our horses and cattle. We must not place the windows close to our horses' heads.

Nothing can have a worse effect upon the eyesight of the horse. Nothing is more annoying to his nervous system than this glaring whitewash and concentrated light directly in front of him. All light should, if possible, come from behind the horse when in the stable. We have known some of the most dangerous shyers cured by obscuring the light in front of the stall and placing it behind. A preparation far better than the shifting, glaring whitewash, is water-lime and skim-milk, with four ounces of alum to the pailful. It has a softer shade, does not rub off, lasts five times as long, and costs but little.

Another point is the fact that all animals, man included, to be healthy and pure, must be properly fed and watered. Bad, malarious water, or water rendered impure from whatever cause, should never be given to our domestic animals, any more than we would partake of such ourselves. As you cannot obtain pure water from an impure spring, even so you cannot obtain pure blood from impure sources, whether of food, water or disease, especially the latter. These last remarks refer chiefly, of course, to our beef, mutton, cheese, butter, milk, etc., but are of equal importance to the horse, with his almost human intelligence, delicate organism and proneness to malarious influences. It is our duty to feed wholesome food and water to all our animals. We shall be amply repaid, for nature never becomes our debtor if we deal fairly and honestly with her

---

A METHOD of procuring fresh water from sea water through the direct action of the sun's rays is among the foreign inventions. The apparatus consists of a box of wood one inch thick, about fourteen feet long, two feet wide, and of an average depth of six inches. The upper part of the box is closed with ordinary glass, which has an inclination of an inch and a half. At the lower edge of the glass there is a semi-circular channel, destined to receive the fresh water which is condensed on the interior surface of the glass. The operation is exceedingly simple. The salt water is let into the box for about an inch in depth, and it is then exposed to the rays of the sun. A very active evaporation begins, and it is found that a square metre of glass will condense daily the amount of two gallons of pure water.

